# Trastuzumab deruxtecan adverse drug reactions reported to the UK’s Medicines and Healthcare products Regulatory Agency via the Yellow Card Reporting System

**DOI:** 10.1016/j.esmoop.2025.105554

**Published:** 2025-08-20

**Authors:** J. Pearson, M. Pirmohamed, C. Palmieri

**Affiliations:** 1 Department of Molecular and Clinical Cancer Medicine, Institute of Systems, Molecular and Integrative Biology, University of Liverpool, Liverpool, UK; 2 The Clatterbridge Cancer Centre NHS Foundation Trust, Liverpool, UK; 3 Centre for Drug Safety Sciences, and Wolfson Centre for Personalised Medicine, Department of Pharmacology and Therapeutics, University of Liverpool, Liverpool, UK

Trastuzumab deruxtecan (T-DXd) is an antibody–drug conjugate targeting human epidermal growth factor receptor 2, whose key life-threatening toxicity is pneumonitis/interstitial lung disease (ILD). A disproportionality analysis in the Federal Drug Administration Adverse Event Reporting System (FAERS) from December 2019 to June 2022 identified 1244 adverse drug reactions (ADRs) where T-DXd was the suspected drug. Of these, 127 were ILD and 52 pneumonitis, with 23 ILD deaths reported.^1^ Given these data and utilising data from the UK Medicines and Healthcare products Regulatory Agency (MHRA) yellow card scheme, we report here the ADRs, nonfatal and fatal, and in particular those related to pneumonitis/ILD (refer to Supplementary Methods, available at https://doi.org/10.1016/j.esmoop.2025.105554).

Between 4 February 2019 and 12 November 2024, a total of 299 spontaneous reports had been submitted to the MHRA, consisting of 771 suspected ADRs. As of 12 November 2024, the ADRs that occurred at a rate of ≥15% by System Organ Class (SOC) were as follows: respiratory [176 (22.8%)], gastrointestinal [159 (20.6%)], and general disorders [159 (20.6%)].

By 12 November 2024, of 176 respiratory ADRs, 118 (67%) were attributable to those grouped under the term ‘report-defined pneumonitis/ILD’ (Supplementary Information, available at https://doi.org/10.1016/j.esmoop.2025.105554). A total of 54 fatal outcomes were reported; 33 of these 54 (61%) deaths were respiratory disorders, with 31 of 54 (57%) being attributable to those grouped under ‘report-defined pneumonitis/ILD’. Of the nonrespiratory fatalities, those that occurred in >5% of patients were reported as death [8 (14.8%)], and disease progression [7 (13.0%)]. Table 1 summarises the cumulative total of suspected ADRs and fatal ADRs.

**Table 1:** Summary of cumulative spontaneously reported suspected adverse drug reactions linked to T-DXd by the Medical Dictionary for Regulatory Activities (MedDRA)

				1 January 2024		24 April 2024		12 November 2024	
Systems organ class	Higher level group term	Higher level term	Preferred term	Total	Fatal	Total	Fatal	Total	Fatal
Respiratory disorders^a^	Lower respiratory tract disorders (excluding obstruction and infection)	Lower respiratory tract inflammatory and immunologic conditions	Pneumonitis^b^	25	8	27	9	36	8
		Parenchymal lung disorders NEC	Acute interstitial pneumonitis^b^	1	1	1	1	1	1
			Interstitial lung disease^b^	38	13	50	15	77	20
			Lung infiltration	0	0	0	0	1	0
			Pulmonary fibrosis^b^	3	1	3	1	3	2
			Pulmonary toxicity^b^	1	0	1	0	1	0
			Restrictive pulmonary disease	0	0	1	0	1	0
		Pulmonary oedema	Acute respiratory distress syndrome	0	0	0	0	2	0
	Bronchial disorders (excluding neoplasms)	Bronchospasm and obstruction	Chronic obstructive pulmonary disease	1	0	1	0	1	0
	Pleural disorders	Pneumothorax and pleural effusions NEC	Pleural effusion	2	0	2	0	4	0
	Pulmonary vascular disorders	Pulmonary thrombotic and embolic conditions	Pulmonary embolism	4	1	3	0	4	0
	Respiratory disorders NEC	Breathing abnormalities	Dyspnoea	10	0	13	0	15	0
			Dyspnoea exertional	1	0	1	0	1	0
		Conditions associated with abnormal gas exchange	Hypoxia	1	0	2	0	2	0
		Coughing and associated symptoms	Cough	9	0	10	0	13	0
			Haemoptysis	0	0	1	0	1	0
		Respiratory failures (excluding neonatal)	Respiratory failure	1	0	2	0	2	0
		Respiratory tract disorders NEC	Lung disorder	2	0	2	0	3	0
			Pulmonary mass	1	1	1	1	1	1
			Respiratory disorder	1	1	1	1	1	1
	Respiratory tract signs and symptoms	Lower respiratory tract signs and symptoms	Lung opacity	0	0	1	0	2	0
		Upper respiratory tract signs and symptoms	Oropharyngeal discomfort	1	0	1	0	1	0
	Thoracic disorders (excluding lung and pleura)	Mediastinal disorders	Pneumomediastinum	2	0	2	0	2	0
	Upper respiratory tract disorders (excluding infections)	Nasal disorders NEC	Epistaxis	0	0	1	0	1	0
	Total classified as pneumonitis/ILD			68	23	82	26	118	31
	Total respiratory not classified as pneumonitis/ILD			36	3	45	2	58	2
All respiratory disorders				104	26	127	28	176	33
Nonrespiratory disorders				285	10	359	13	595	21^c^
Total				389	36	486	41	771	54

Suspected adverse drug reactions (ADRs) were classified by the Medical Dictionary for Regulatory Activities (MedDRA) Version 26.1 for the January 2024 cut-off, and 27.1 for the April and December cut-off, where they were grouped by System Organ Class (SOC) based on aetiology and site of toxicity. From here they were coded into ‘High Level Group Terms’ and ‘High Level Terms’, based upon anatomy, physiology and pathology, and then ‘Preferred Terms’ as a distinct descriptor by symptom, sign or disease diagnosis.

ILD, interstitial lung disease; NEC, not elsewhere classified.

a This was referred as ‘Respiratory, thoracic and mediastinal disorders’ in version 27.1.

b Classified as pneumonitis/ILD.

c Summary of 21 nonrespiratory causes of death by SOC and preferred term: General disorders: Death (8 of 21); Disease progression (7 of 21); General physical health deterioration (1 of 21); Blood and lymphatic system disorders: Thrombocytopaenia (1 of 21); Gastrointestinal: Colitis (1 of 21); Infections and infestations: Sepsis (1 of 21); Investigations: Ejection fraction decrease (1 of 21); Neoplasms benign, malignant and unspecified (incl cysts and polyps): Breast cancer metastatic (1 of 21).

The current data demonstrate that ADRs and fatal ADRs were most commonly respiratory, with 57% of all deaths related to the preferred terms: pneumonitis, acute interstitial pneumonitis, ILD, pulmonary toxicity, and lung fibrosis. While FAERS found 23 deaths from ILD/pneumonitis from 1244 ADRs reported,^1^ MHRA data revealed 31 deaths from 771 reported ADRs, utilising report-defined pneumonitis/ILD. Real-world data from Japan revealed 287 of 3000 (9.6%) patients treated with T-DXd developed potential T-DXd-related ILD/pneumonitis.^2^ Of those that underwent subsequent adjudication, the mortality rate was 11 of 130 cases (8.5%).^2^ Data from France has reported rates of 3.7% (*n* = 459) with no deaths related to pneumonitis/ILD.^3^

Our data are limited, given that the total number of patients treated with T-DXd is not known, preventing direct comparisons with other data; the absolute number of deaths in our study, however, is higher. The MHRA reports that only 10% of ADRs are reported to it,^4^ and thus the current data could be an underrepresentation of the true picture. Additionally, these data are derived from spontaneous reports with the ADRs attributed by the reporter, and so causality cannot be certain and may instead reflect other pathological processes. This limitation is common to all spontaneous ADR reporting schemes, including the FAERS data. Whether the higher death rate in the MHRA data is accurate or whether it is an anomaly of the spontaneous reporting systems is unclear. It is important for prescribers to be aware of this serious ADR, and follow the guidance provided in the drug label in terms of monitoring and drug discontinuation.

In summary, these UK data provide further real-world data about T-DXd and ILD/pneumonitis. They demonstrate that the respiratory system is the most commonly affected organ system both in terms of total and fatal ADRs. The current data underline the vital importance of postmarketing surveillance once a new agent is licenced.
