# Supplementary material for: Trastuzumab deruxtecan adverse drug reactions reported to the UK’s Medicines and Healthcare products Regulatory Agency via the Yellow Card Reporting System
Source: ESMO Open. 2025 Aug 20;10(9):105554. doi: 10.1016/j.esmoop.2025.105554 (PMC12529297; doi:10.1016/j.esmoop.2025.105554)
Supplement: Supplementary Material [file mmc1.docx]

**Trastuzumab deruxtecan adverse drug reactions reported to the United Kingdom’s Medicines and Healthcare products Regulatory Agency via the Yellow Card Reporting System**

James Pearson^1,2^, Munir Pirmohamed^3^, Carlo Palmieri^1,2^

**Supplementary methods**

The MHRA collect all the UK’s spontaneous suspected adverse drug reactions (ADRs) reports, which are submitted via the Yellow Card Scheme (<https://yellowcard.mhra.gov.uk/>), on behalf of the UK Government’s Commission on Human Medicine independent scientific committee. Suspected ADRs are reported on a voluntary basis when a healthcare professional has a suspicion that the medicine has caused the reaction.

Data was requested from the MHRA on spontaneous ADRs reported in relation to T-DXd, and data was made available for the period covering the 4th February 2019 to 4th November 2024. For the purpose of this report, the following preferred terms: pneumonitis, acute interstitial pneumonitis, ILD, pulmonary fibrosis, and pulmonary toxicity were grouped together under the term ‘report-defined pneumonitis/ILD’.

Three requests were made on the 19th December 2023, 19th April, and 9th September 2024, with reports returned with respective data cuts as follows: 1st January, 24th April, and 12th November 2024. The data provided covers the period 4th February 2019 to 4th November 2024.

Suspected ADRs were classified by the Medical Dictionary for Regulatory Activities (MedDRA) Version 26.1 for the January 2024 cut off, and 27.1 for the April and December cutoff, where they were grouped by System Organ Class (SOC) based on aetiology and site of toxicity. From here they were coded into “High Level Group Terms” and “High Level Terms”, based upon anatomy, physiology and pathology, and then “Preferred Terms” as a distinct descriptor by symptom, sign, or disease diagnosis.
